# Assessing Polymer-Surface Adhesion with a Polymer Collection

**DOI:** 10.1021/acs.langmuir.1c02724

**Published:** 2022-02-09

**Authors:** Stephan Eickelmann, Sanghwa Moon, Yuxin Liu, Benjamin Bitterer, Sebastian Ronneberger, Dominik Bierbaum, Frank Breitling, Felix F. Loeffler

**Affiliations:** † Max-Planck-Institute of Colloids and Interfaces, Biomolecular Systems, Am Muehlenberg 1, 14476 Potsdam, Germany; ‡ Institute of Microstructure Technology, Karlsruhe Institute of Technology, Hermann-von-Helmholtz-Platz 1, 76344 Eggenstein-Leopoldshafen, Germany

## Abstract

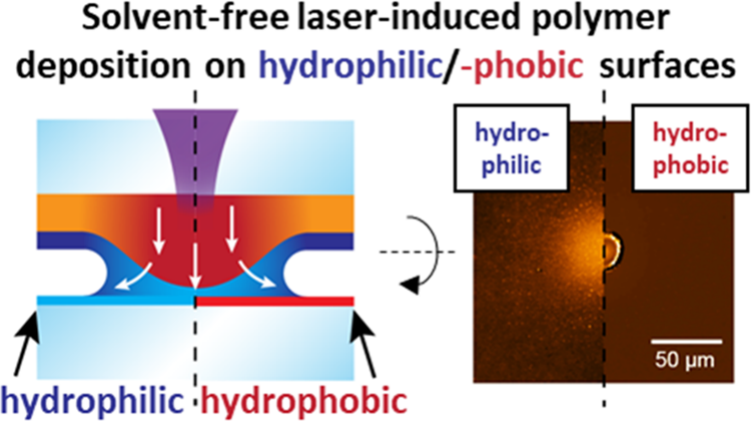

Polymer modification plays an important role in the construction of devices, but the lack of fundamental understanding on polymer-surface adhesion limits the development of miniaturized devices. In this work, a thermoplastic polymer collection was established using the combinatorial laser-induced forward transfer technique as a research platform, to assess the adhesion of polymers to substrates of different wettability. Furthermore, it also revealed the influence of adhesion on dewetting phenomena during the laser transfer and relaxation process, resulting in polymer spots of various morphologies. This gives a general insight into polymer-surface adhesion and connects it with the generation of defined polymer microstructures, which can be a valuable reference for the rational use of polymers.

## 1 Introduction

Inert polymer coating is one of the most widely used methods for surface engineering in the construction of functional devices.^1−4^ It can not only protect the devices from a harsh environment but also provide a facile and efficient way to modulate their hydrophilic–hydrophobic nature to meet the requirements of diverse applications in biomedical and engineering fields.^5−8^

Recent trends of integration and portability generate growing demand for miniaturized devices, which brings great opportunity to this field. However, the uncertainty of the polymer-surface adhesion slows down this evolution.^9,10^ On the one hand, spontaneous dewetting phenomena of polymers on substrates make it hard to generate polymer microstructures with defined morphologies (e.g., size, thickness, and shape).^11−17^ On the other hand, mismatched polymer–substrate pairs lead to poor adhesion, having a negative impact on device stability. Therefore, it is necessary to get a better control of polymer-surface adhesion. A typical method is to introduce secondary bonds on the interface between the polymer and substrate (e.g., causing hydrogen bonds or host–guest interaction), resulting in relatively stronger adhesion than conventional van der Waals interaction. In addition, recent advances also showed the possibility to control the adhesion with an external stimulus (e.g., light or heat) by modulating void spaces and surface gaps at the polymer–substrate interface.^18−20^ While many studies were committed to find effective ways to control adhesion, it remains an essential goal to develop a fundamental understanding of adhesion, especially in the microscale region.

**Figure 1:**
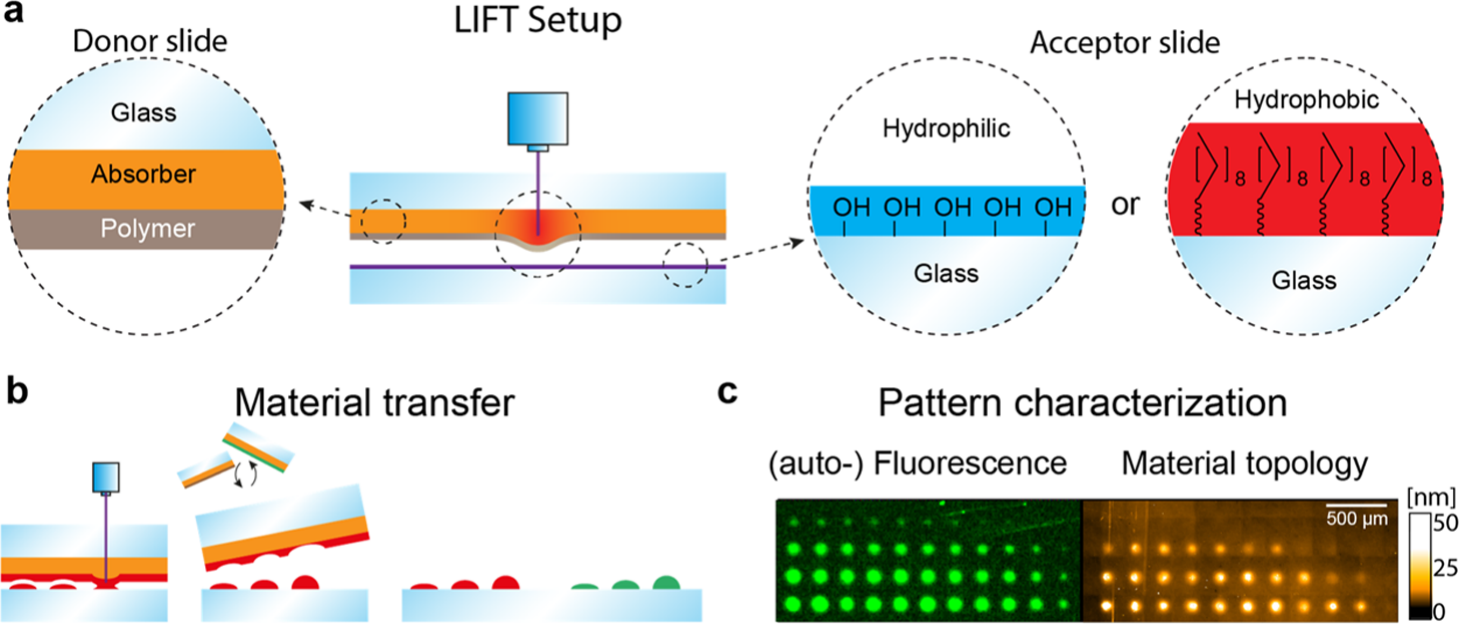
(a) cLIFT principle, transferring different thermoplastic polymers with a laser onto hydrophobic/hydrophilic surfaces. (b) Pattern generation process via the laser scanning system and (c) characterization via fluorescence scan and vertical scanning interferometry.

Laser-induced forward transfer (LIFT) is a versatile mask-free method to generate thin-film surface patterns of almost any material and is widely applied in micro-device fabrication.^21^ The recently developed combinatorial LIFT (cLIFT) technique is an emerging cost-efficient technique for micro-device construction since it relies on simple diode lasers and can be used to generate polymer patterns easily and rapidly without the typical limitations of solvents.^22,23^ Under laser irradiation, a certain amount of thermoplastic polymer at the focused spot is heated, melted, and then precisely transferred to an acceptor slide within milliseconds (Figure 1a). Notably, by manipulating the laser irradiation parameters (e.g., spot size, power density, and irradiation time), the amount of the transferred polymer can be modulated, which is ideal to observe the behavior of polymer microdrops with controllable volume on a surface (Figure 1b).^24^ Therefore, the cLIFT technique is not only a representative method for the construction of miniaturized devices but a useful tool to study polymer-surface adhesion in the microscale region.

Herein, a series of commercial thermoplastic polymers were studied on both hydrophobic and hydrophilic surfaces. The morphologies of formed polymer micropatterns were collected to reveal the influence of polymer polarity and surface wettability on the polymer-surface adhesion. This could be utilized as a reference to guide the use of thermoplastic polymers and the exploration of new polymers for surface engineering.

## 2 Experimental Section

### 2.1. Laser Transfer of Polymer Spots

The system operates with a 488 nm diode laser with a peak power output of around 120 mW, focused to about 17 μm in diameter (Supporting Information Figure S1). In brief, the laser is controlled by an ARGES Raccoon 11 laser scanning system, focused through an f-Theta lens onto the substrate (details can be found in a study by Mende et al.^25^).

### 2.2 Acceptor Preparation

The substrates were sonicated in an ultrasound bath for 5 min each in a sequence of double-distilled water (ddH_2_O), isopropanol, acetone, isopropanol, and ddH_2_O. Then, they were cleaned with piranha solution [3:1 (v/v)—H_2_SO_4_/H_2_O_2_] for ∼30 min@70 °C to expose the hydroxyl groups. Afterward, they were flushed with copious amounts of water, sonicated again in ddH_2_O for about ∼10 min, and finally stored in ddH_2_O for a maximum of 6 h until use. Directly before use, they were rinsed again with fresh ddH_2_O and dry blown with clean nitrogen (purity 5.0).

### 2.3 Silanization

The surfaces of the glass were hydrophobized by coating pure monolayers of alkyl-chlorosilanes to the hydroxyl groups via chemical vapor deposition. The coating was performed in a standard desiccator under constant evacuation (residual pressure ∼50 mbar). Before evacuation, the samples were placed in a Petri dish near its perimeter. In the center, a small vessel provided a single liquid drop (∼20 μL) of the dimethyl-decyl-chlorosilane (ABCR, Karlsruhe, Germany, purity 97%, used as obtained). The samples were kept under vacuum (under constant evacuation ∼50 mbar) together with the drop of silane for ∼3 h. As described in the literature,^26^ the exact process time does not have a significant effect on silanization. After gas-phase salinization, the samples were annealed for about 1 h at 100 °C under a nitrogen atmosphere. Finally, glass slides were sonicated for 10 min in hexane.

### 2.4. Donor Slide Preparation

The commercially available Kapton HN tape (DuPont, USA; cmc Klebetechnik GmbH, Germany; thickness of the polyimide layer approx. 25 μm and thickness of the glue layer approx. 35 μm) was laminated onto 1 mm thick microscopy glass slides. Then, the polymer films were prepared as follows: 40 mg of the polymer was dissolved in 1000 μL of dichloromethane (DCM), or for the water-soluble polymers, 25 mg in 1000 μL of water (Supporting Information Figure S2a). The mixtures were spin-coated at 80 rps onto the polyimide film, resulting in 100–200 nm film thickness (according to a study by Danglad-Flores et al.^27^). An overview of additionally used polymers can be found in the Supporting Information (Table S1).

### 2.5 Vertical Scanning Interferometry

High-magnification (50× & 100× Nikon CF IC Epi Plan DI Mirau) vertical scanning interferometry (VSI) was performed with a smartWLI compact (Gesellschaft für Bild-und Signalverarbeitung mbH, Ilmenau, Germany), and the images were stitched together via software MountainsMaps (Digital Surf, France).

### 2.6 Fluorescence Scanning

Fluorescent image acquisition was performed with the fluorescence scanner Genepix 4000B (Molecular Devices, USA) at the wavelengths 532 and 635 nm with a laser power of 33%, a resolution of 5 μm, and a photo multiplier gain of 600.

### 2.7 Differential Scanning Calorimetry

Thermal properties were studied via differential scanning calorimetry (DSC) with a DSC 2500 (TA Instruments, USA) under a nitrogen atmosphere using about 5 mg of the respective sample. The polymers were heated with a heating and cooling rate of 10 °C/min. Glass-transition points were determined by calculating the half height of the step transition and melting points as peak temperature of the second heating scan.

### 2.8 Thermogravimetric Analysis

Thermogravimetric analysis was performed on a TGA 5500 instrument (TA-Instruments, USA) using about 10 mg of the polymer in HT platinum pans from 40 to 700 °C and a heating rate of 10 K/min under a nitrogen atmosphere.

## 3 Results and Discussion

### 3.1 Optimization of Laser Parameters

To investigate the laser transfer of different thermoplastic polymers, we used a collection of commercially available thermoplastic polymers (Tables 1 and S1), including polyvinyl chloride (PVC), polylactic acid (PLA), polyvinyl alcohol (PVA), three different poly(styrene-*block*-methyl methacrylate) copolymers (PS-*b*-PMMA 21 k:21 k, 20 k:50 k, and 52 k:52 k), and styrene–butylacrylic copolymer (SLEC). These polymers were mainly selected due to standard availability and solubility in organic solvents, such as DCM and toluene, and only PVA is solely soluble in water. Besides the standard homopolymers, two types of copolymers were used: SLEC, a styrene-acrylic copolymer typically used in our LIFT variants, and three different PS-*b*-PMMA block-copolymers with different polarity but very similar physical and chemical properties.

**Table 1:**
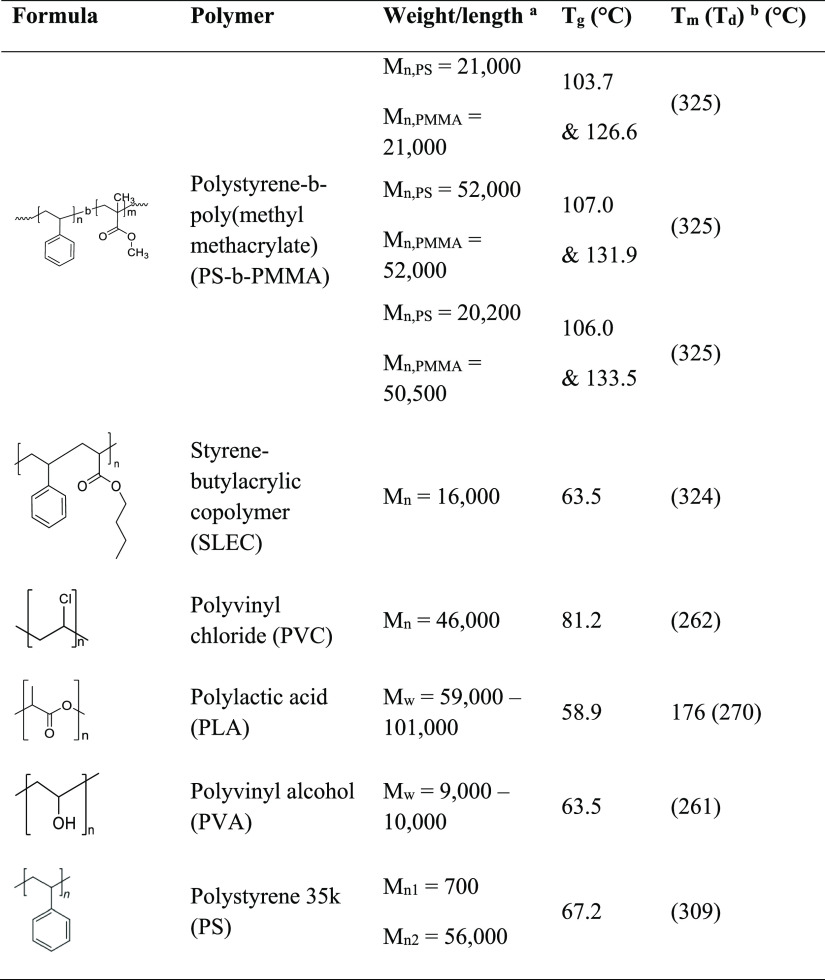
Studied Thermoplastic Polymers and Related Physical Properties

a In *M*_w_ or *M*_n_.

b Melting and decomposition temperatures, *T*_d_ (5%), are presented in brackets.

Although being commercially available, not all vendors provide sufficient information on the polymers. Therefore, we determined the glass-transition temperature with DSC and the decomposition temperature with thermogravimetric analysis.

The glass-transition temperatures vary between −60 and 135 °C (including Supporting Information Table S1), while all decomposition temperatures are above 250 °C. Due to the *block*-copolymer character, the PS-b-PMMAs feature two glass-transition temperatures, one for each block. According to our measurements and the vendor information, the two polymers PLA and PCL 14 k (Supporting Information Table S1) are semicrystalline, and we observed a melting temperature *T*_m_.

**Figure 2:**
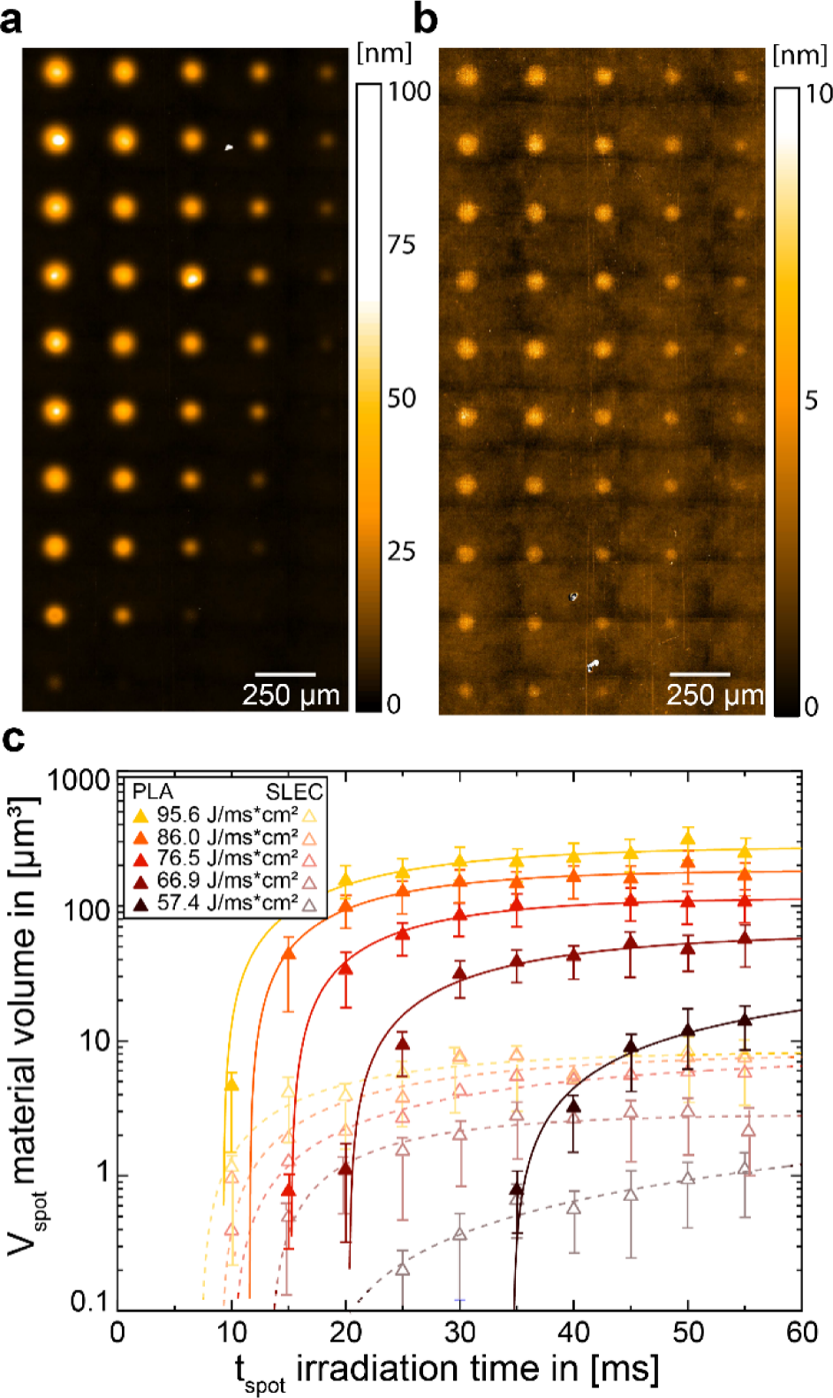
VSI measurements of transferred gradient patterns of (a) PLA and (b) polystyrene butylacrylic copolymer (SLEC) on a hydrophilic glass surface. (c) Material volume for different power values (different colors) is shown as a function of irradiation time, with exponential fits.

Next, to find optimum parameters for the laser transfer, a laser energy gradient pattern was defined in the LIFT system. We varied the laser energy from 57.4 to 95.6 J ms^–1^ cm^–2^ in increments of 9.5 J ms^–1^ cm^–2^ along the short axis and the irradiation time from 5 to 55 ms in 5 ms increments on the long axis, with a spacing of 250 μm between the spots (Figure 2a,b). After the transfer of several polymer microarrays onto an acceptor slide, the polymer patterns were analyzed with VSI, to evaluate the polymer spot morphology, and auto-fluorescence measurements, to detect trace amounts of material (<1 nm), which may not be visualized in VSI (Figure 1c).

**Figure 3:**
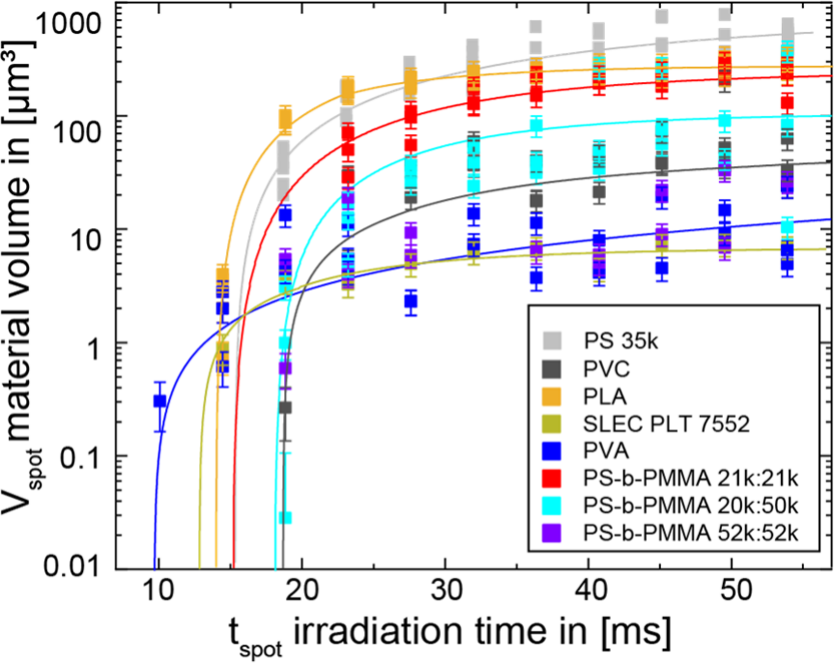
Material volume of several different polymers for a laser power of 95.4 J ms^–1^ cm^–2^ is shown as a function of irradiation time with exponential fits. For improved visualization, only the data from maximum laser power over irradiation time were plotted.

As a first example, the transfer of PLA and polystyrene acrylic copolymer (SLEC) in gradients onto a clean glass substrate was analyzed with VSI (Figure 2a,b. For each spot, the material volume was determined and plotted as a function of the irradiation time for the five different power values (Figure 2c). Both polymers show increasing amounts of transferred material with increasing irradiation time, whereas the PLA spots generally show a higher amount of transferred material. The behavior of these polymers is similar, and the transferred material amounts seem to follow an exponential behavior in both cases. While at lower irradiation times, the material amounts increase with increasing irradiation time, and a plateau is obtained for higher irradiation times. Apart from PLA and SLEC, other polymers (PVC, PVA, and three PS-*b*-PMMAs) were studied in a similar manner (Figure 3).

### 3.2 Influence of Polymer Polarity

All selected polymers could be transferred as spot patterns (Supporting Information Figures S3–S5). However, not all polymer spots offered sufficient material thickness for VSI analysis. For those polymers, only the auto-fluorescence scan indicated transfer of a sub-nanometer thin layer of the polymer. Neither the characteristic glass transition, nor the melting temperatures and the molecular weight of the polymers showed a direct correlation with the transferred spot volume. Some polymers offer a large range of transferred material volume, for example, PLA. From our data, for each polymer, we found an individual lasing parameter space for a desired optimal transfer. In addition, also large polymer patterns can be generated (Supporting Information Figure S7), making the approach useful for the generation of devices, for example, anti-counterfeiting labels.^28^

**Figure 4:**
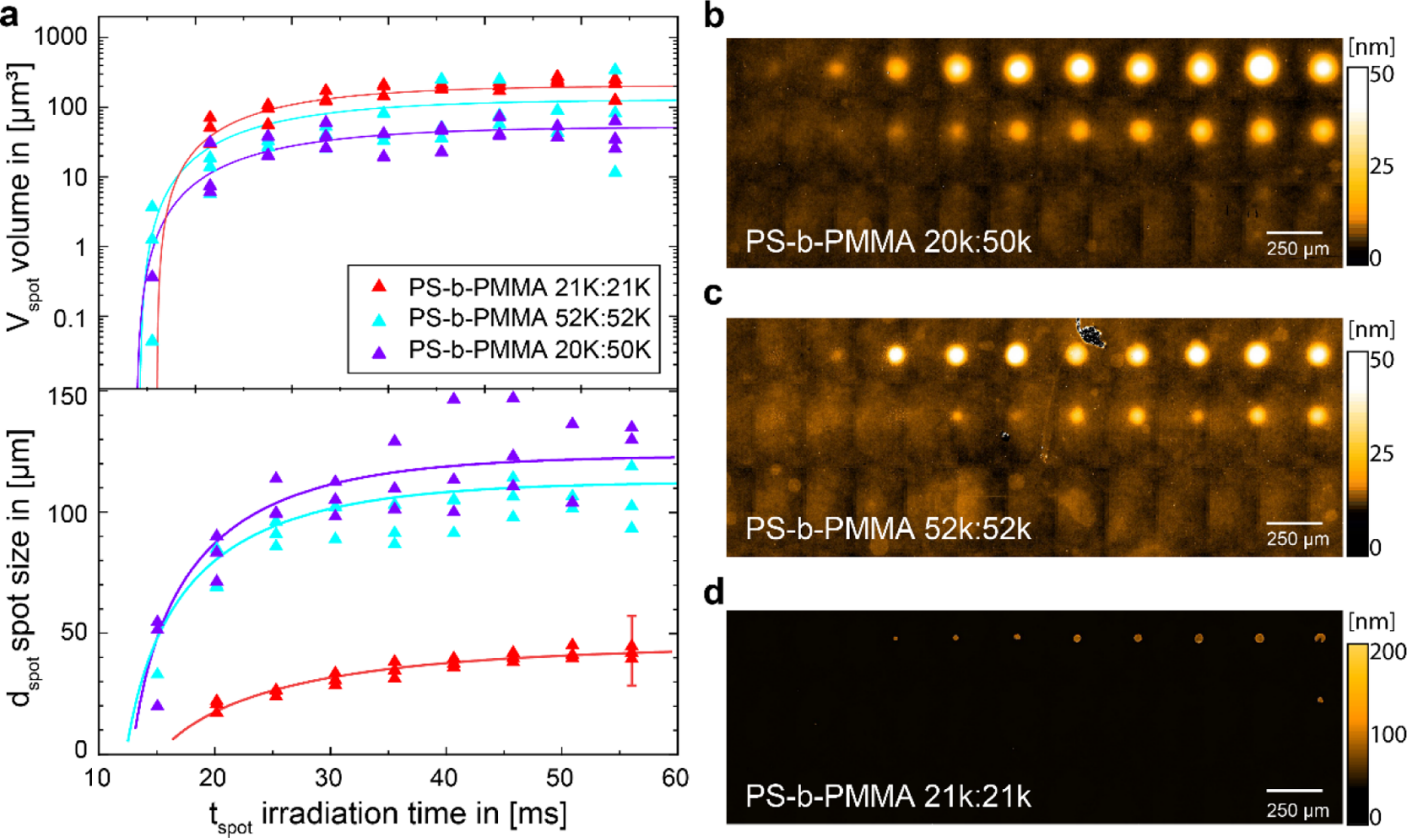
(a) VSI measurements show the material volume vs spot diameter as a function of irradiation time for a laser power of 95.4 J ms^–1^ cm^–2^. Corresponding VSI measurements of (b) PS-*b*-PMMA 20 k:50 k, (c) PS-*b*-PMMA 52 k:52 k, and (d) PS-*b*-PMMA 21 k:21 k. Error bar only shown for one value for clarity.

Interestingly, for the three block copolymers, the transferred material volumes were very similar, while their spot morphologies differed significantly. The three different PS-*b*-PMMA block-copolymers have similar material properties, except for their wetting behavior due to different block lengths.^29,30^ PMMA is relatively more hydrophilic, than PS and it has been shown that PS-PMMA copolymers become more hydrophobic with increasing PS contents.^31^ PS-*b*-PMMA films are well known to form self-assembled nanostructures of PS and PMMA domains upon heating, such as islands or lamellas. Depending on the length of the polymer blocks, they can form lamellar nanostructures of different sizes, for example, ∼10 nm lamella spacing for PS-*b*-PMMA 25–26 k and ∼50 nm lamella spacing for PS-*b*-PMMA 52–52 k.^30^ While two of our block copolymers (21 k:21 k and 52 k:52 k) have a similar PS/PMMA ratio, the different nanostructures will influence the wettability properties of the copolymers. The larger PMMA domains in PS-*b*-PMMA 52 k:52 k should cause a higher polarity than that in PS-*b*-PMMA 21 k:21 k, whereas PS-*b*-PMMA 20 k:50 k should be the most polar copolymer. Therefore, we measured the volume, as well as the diameter of the laser-transferred material spots as a function of the irradiation time (Figure 4).

While the volume of PS-*b*-PMMA 21 k:21 k and PS-*b*-PMMA 52 k:52 k is quite similar (PS-*b*-PMMA 20 k:50 k gave smaller amounts), the spot diameter is much smaller for the PS-*b*-PMMA 21 k:21 k. Here, PS-*b*-PMMA 20 k:50 k and PS-*b*-PMMA 52 k:52 k show similar spot diameters, while the PS-*b*-PMMA 21 k:21 k forms small spot diameters. VSI measurements of the three block-copolymers show that while for the more polar PS-*b*-PMMA 52 k:52 k and PS-*b*-PMMA 20 k:50 k, large but flat spots can be observed, and the rather non-polar PS-*b*-PMMA 21 k:21 k features small spot diameters with a much larger height.

### 3.3 Influence of Surface Wettability

To further study the influence of the surface wettability of the block-copolymers, we prepared hydrophilic and hydrophobic slides by two different surface modification methods, respectively (Supporting Information Figure S2b).

**Figure 5:**
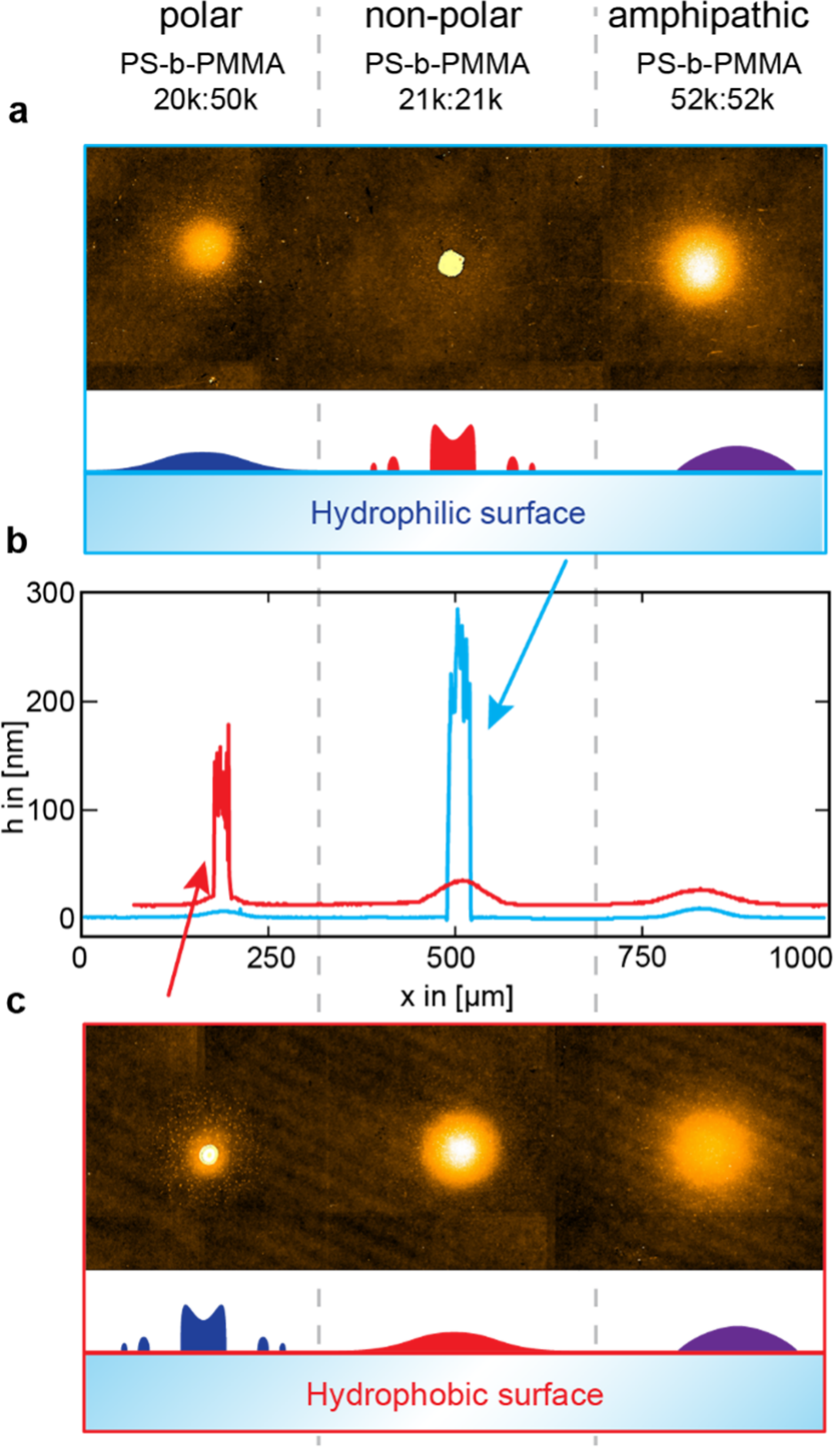
VSI measurements of spots of three different block-copolymers, PS-*b*-PMMA 20 k:50 k, 21 k:21 k, and 52 k:52 k, transferred with the same lasing conditions (55 ms at 60.7 J ms^–1^ cm^–2^) onto (a) hydrophilic and (c) hydrophobic glass. Illustrations depict the observed spot morphologies. (b) Height profile of all three spots on the hydrophobic (red) and the hydrophilic surface (blue). For volumetric details, see Supporting Information Figure S6.

The transfer of a polar polymer onto a hydrophobic surface results in a very steep spot with obvious dewetting artefacts like a rim formation and a very high contact angle (Figure 5a,b). On the same surface, the much less polar counterpart creates a very smooth spot with a low contact angle.

The analogous experiment on a hydrophilic surface results in the opposite behavior (Figure 5b,c). The polar polymer creates a very homogeneous flat spot, while the hydrophobic one creates a small and very sharp spot with dewetting artefacts.

The amphiphilic polymer without any clear wetting favor forms a similar morphology on both surface modifications. From our previous work, we observed that the laser induces a thermal expansion inside the polyimide film.^24^ This leads to a deformation of the surface and eventually to a contact with the acceptor, which results in a material transfer. During the thermal expansion of the polyimide, also the polymer coating on the surface starts to heat up and softens/melts. The softened/molten polymer is pressed against the acceptor surface. Once the laser irradiation stops, the polyimide starts to cool, recedes, and detaches from the acceptor, while parts of the polymer will remain on the acceptor.

From this mechanism, we deduce that changing to a different polymer coating should not have a strong influence on the contact-based transfer mechanism. However, the softening and melting of the polymer should strongly influence the material deposition. Most of the thermoplastic polymers have similar glass-transition temperature ranges. From our previous studies, where we determined the maximum temperatures and temperature profiles achieved during the laser transfer,^32^ we determined a maximum temperature between 200 and 500 °C with a quickly diminishing thermal gradient around the laser focus. In its center, the decomposition temperature of the polymer may be reached. However, since the process is localized and very fast (in milliseconds), the polymer appears unharmed.

**Figure 6:**
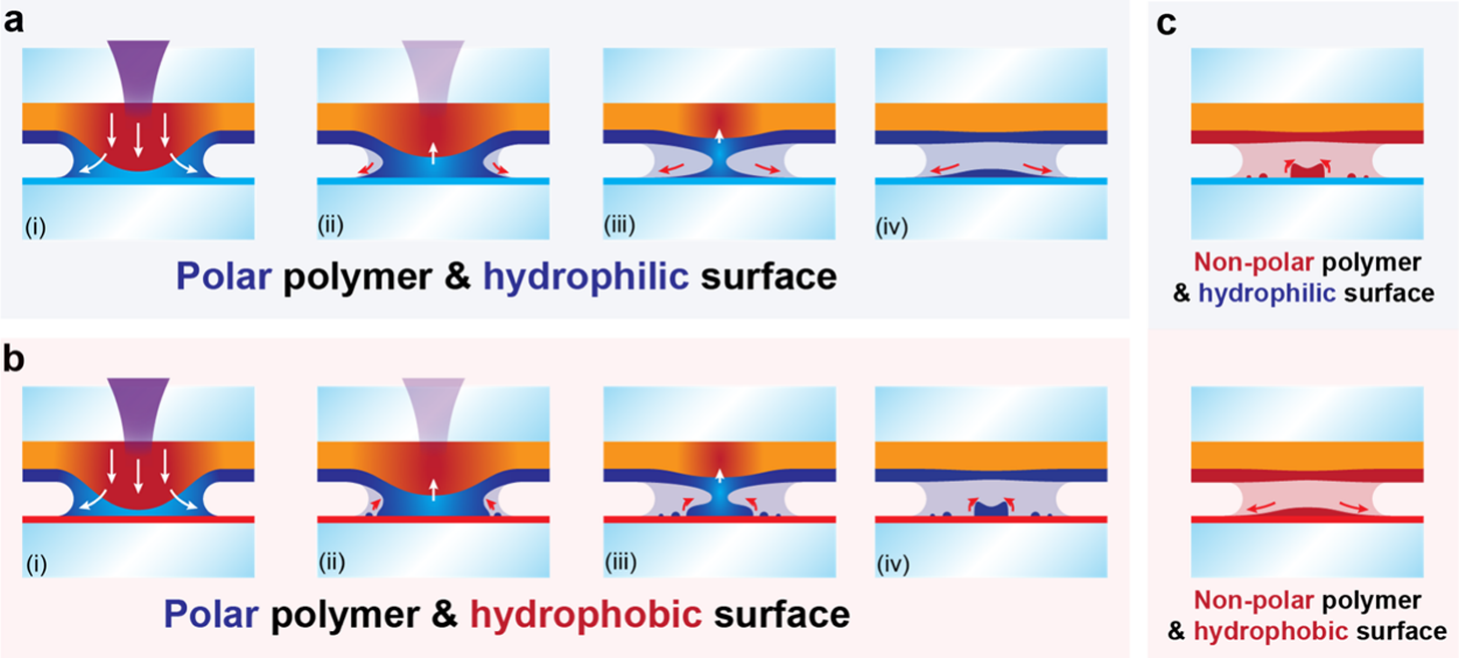
Mechanism of the contact transfer for different surface wettability properties. Polar polymer transferred onto (a) hydrophilic or (b) hydrophobic glass. (c) For a non-polar polymer, the wetting behavior is inverted.

Since the transfer mechanism should be similar for different polymers, we investigated the adhesion of the polymer with the acceptor surface, which may cause different spot morphologies and sizes. Based on these findings, we have developed a hypothesis for the transfer mechanism on hydrophilic/hydrophobic surfaces (Figure 6).

If a polar polymer is transferred onto a hydrophilic acceptor (Figure 6a), the molten polymer wets the interface during the contact phase of the transfer, while during relaxation, a neck forms, and the polymer will solidify simultaneously. Eventually, the neck will detach, and some material remains in a thin droplet shape on the accepting surface. In the second case, if a polar polymer is transferred onto a hydrophobic surface (Figure 6b), the initial laser-induced contact is similar, whereas due to the mismatch in wettability, parts of the polymer film destabilize and cause the spot to shrink, yielding small residuals on the acceptor surface. Adhesion is high enough to not cause complete removal of the material but low enough to cause dewetting. While the polyimide relaxes, a neck forms, and at the same time, the polymer continues to dewet on the surface, until the final morphology is “frozen” into place. The material on the acceptor forms a much smaller spot with a dewetting rim (elevated edges) and fine residual drying marks. In both cases, even after the transfer process has finished, the material spots slowly continue to dewet over hours or days.^33^ In the reverse case (Figure 6c), the analogous behavior is observed.

Thus, the amount of material, which is transferred in a spot, is determined by the contact-based process, while the spot shape and especially the spot diameter is determined by the wetting properties of the surface and the polymer.

To support our conclusions, we also transferred polymers onto two different monocrystalline silicon substrates, one with a thin ∼5 nm silicon oxide layer (Siegert Wafer, Germany, relatively more hydrophilic) and one with a thicker ∼300 nm silicon oxide layer (Silchem, Germany, relatively more hydrophobic). However, due to the strong light reflection and interference effects of these silicon surfaces (Supporting Information Figure S8), it is impossible to observe the polymer patterns on the silicon substrate by VSI or fluorescence scanning, which we previously used for the characterization of the glass substrates. Other analysis techniques, such as atomic force microscopy, are too slow and limited in the field of view. However, to acquire a qualitative result, carbon black was added to the polymer as a contrast agent for VSI (Supporting Information Figure S8). The polar PVA formed larger spots on the hydrophilic than that on the hydrophobic silicon substrate, which is in accordance with our previous observations.

Finally, we investigated the effect of donor slide polymer layer thickness on the transferred spot height. By spin-coating different polymer concentrations, we generated two different PS layer thicknesses (300 and 700 nm) on donor slides and used them to transfer spot gradient patterns onto a glass substrate (Supporting Information Figures S9 and S10). While the transfer from the thinner PS layer resulted in thinner spots (up to 5 nm), the transfer from the thicker PS layer resulted in thicker spots (up to 10 nm) for the same laser parameters. Thus, a thicker polymer layer allows transferring more material. However, since thicker layers also require more concentrated polymer solutions for spin-coating, the donors have more coating defects, resulting in a partially unstable pattern transfer. Therefore, we restricted the polymer concentrations to experimentally observe homogeneous spin-coating films and reproducible patterns.

## 4 Conclusions

In summary, we studied the polymer-surface adhesion in the microscale region using the cLIFT technique to generate micropatterns of a series of commercial thermoplastic polymers. It was observed that, by adjusting the polymer polarity and acceptor surface wettability, the adhesion could be modulated. During the heat-induced laser transfer and its relaxation process, the polymers showed different dewetting behavior on the substrates and formed various residual microstructures. Besides, depending on polymer polarity and acceptor surface wettability, the polymer-surface adhesion in the microscale region could be indirectly recorded. In future investigations, besides the wetting behavior of the polymers, also the thickness of the LIFT-transferred polymer should be further investigated, which is important for potential device development. By providing a solvent-independent reference, our approach will not only guide the rational use of currently available polymers for device generation but also it will be of interest for the development of next-generation polymers for surface engineering..

## Supporting Information Available

Additional experimental results and details, including the laser profile, functionalization methods, and additionally used polymers (PDF)
