# Supplementary material for: Assessing Polymer-Surface Adhesion with a Polymer Collection
Source: Langmuir. 2022 Feb 9;38(7):2220–6. doi: 10.1021/acs.langmuir.1c02724 (PMC8867722; doi:10.1021/acs.langmuir.1c02724)
Supplement: Supplementary file 1 — la1c02724_si_001.pdf [file la1c02724_si_001.pdf]

Supporting Information

Assessing Polymer-Surface Adhesion with a Polymer Collection

Stephan Eickelmann[1]‡, Sanghwa Moon[1]‡, Yuxin Liu[1], Benjamin Bitterer[2], Sebastian Ronneberger[1], Dominik Bierbaum[1], Frank Breitling[2], Felix F. Loeffler[1]*

[1]Max-Planck-Institute of Colloids and Interfaces, Biomolecular Systems, Am Muehlenberg 1, 14476 Potsdam, Germany
[2]Institute of Microstructure Technology, Karlsruhe Institute of Technology, Hermann-von-Helmholtz-Platz 1, 76344 Eggenstein-Leopoldshafen, Germany
*Email: felix.loeffler@mpikg.mpg.de

This file includes:

Figure S1. Laser power density distribution

Figure S2. Donor and acceptor slide preparation

Figure S3. Fluorescence scan *vs.* topological VSI scan (SLEC)

Figure S4. Fluorescence scan *vs.* topological VSI scan (PLA)

Figure S5. Various polymer transfers

Figure S6. Individual VSI images of spots of the three block-co-polymers

Figure S7. Fluorescence scan of transferred macroscopic polymer patterns

Figure S8. VSI measurements on silicon substrates

Figure S9. Spot gradient on glass, transferred from a thin polystyrene donor

Figure S10. Spot gradient on glass, transferred from a thick polystyrene donor

Table S1. Overview of additional thermoplastic polymers

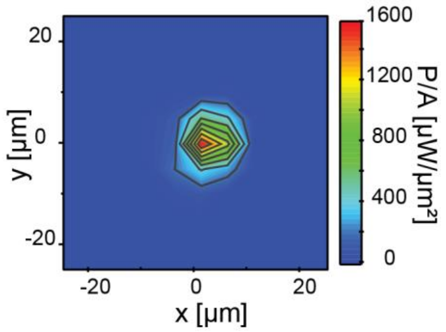

Figure S1. Laser power density distribution in the laser spot at 100% intensity (95.6 J ms^{-1} cm^{-2}).

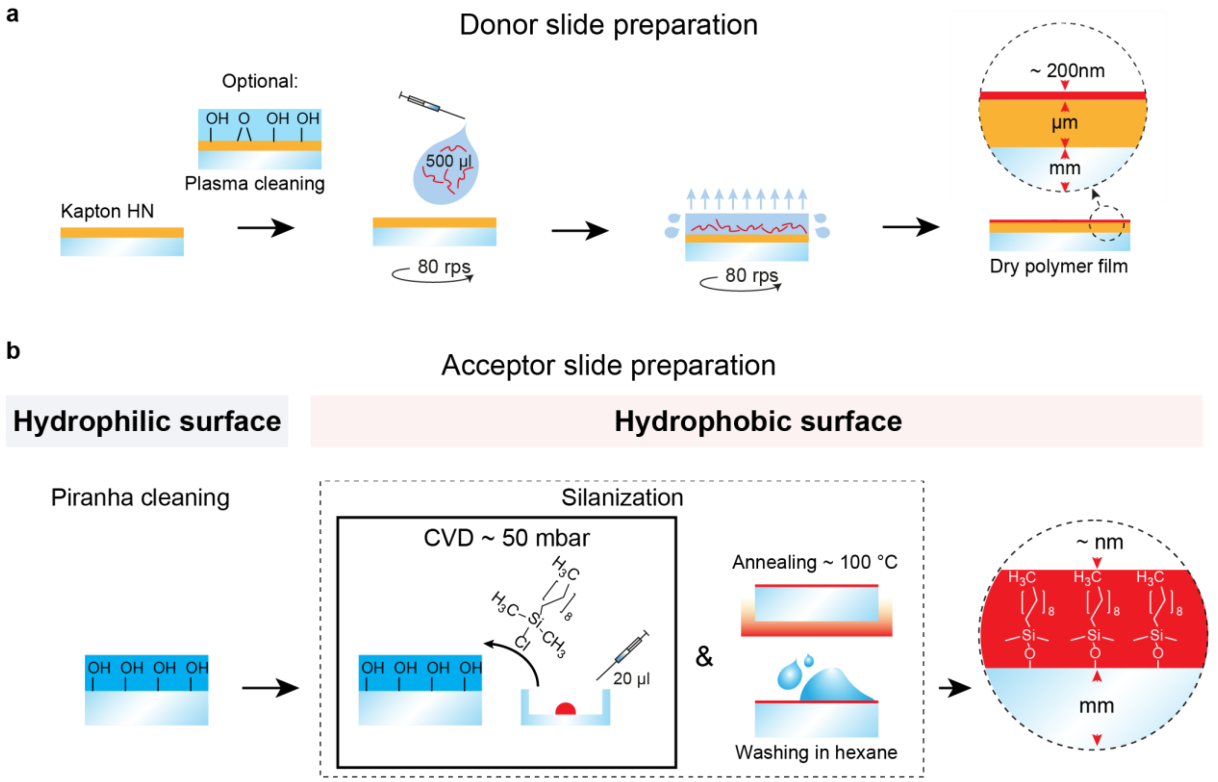

Figure S2. (a) Donor slide preparation *via* spin coating. (b) Acceptor slide surface modification *via* piranha cleaning (hydrophilic) and subsequent silanization (hydrophobic).

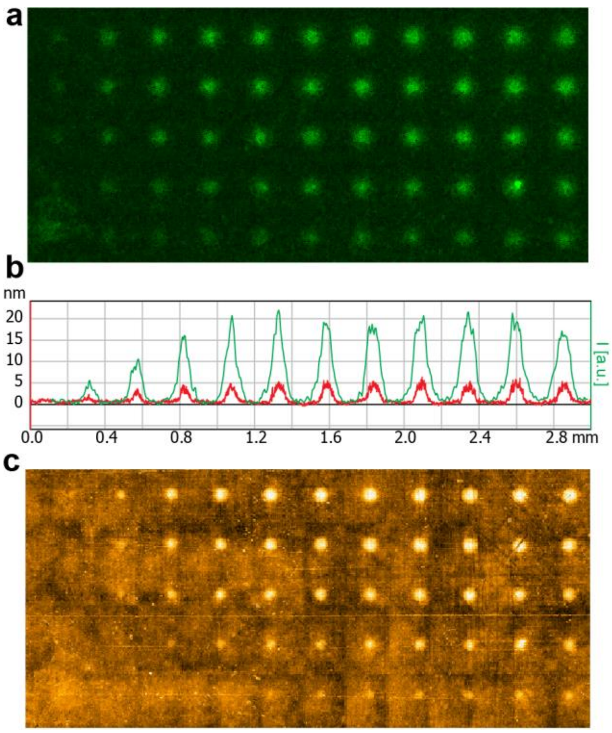

Figure S3. Difference between fluorescence scan (a) and topological VSI Scan (c) of SLEC on a hydrophilic surface. (b) Comparing the cross sections through the maximum power pattern (top line) shows the sensitivity for molecular thin material deposits on the surface, which are not visible in the VSI scan. Spot widths differ significantly in the two measurement approaches.

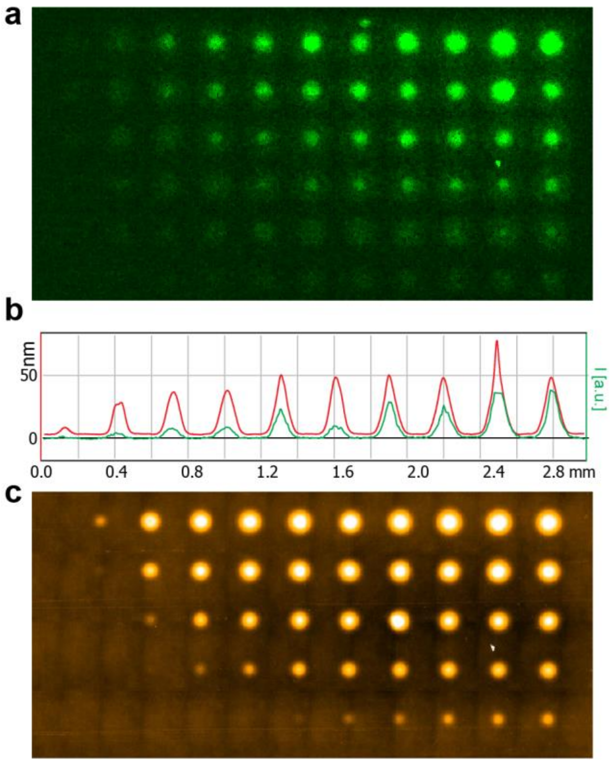

Figure S4. Difference between fluorescence scan (a) and topological VSI scan (c) of PLA on a hydrophilic surface. (b) Comparing the cross sections through the maximum power pattern (top line) shows that the spot widths match in both measurements.

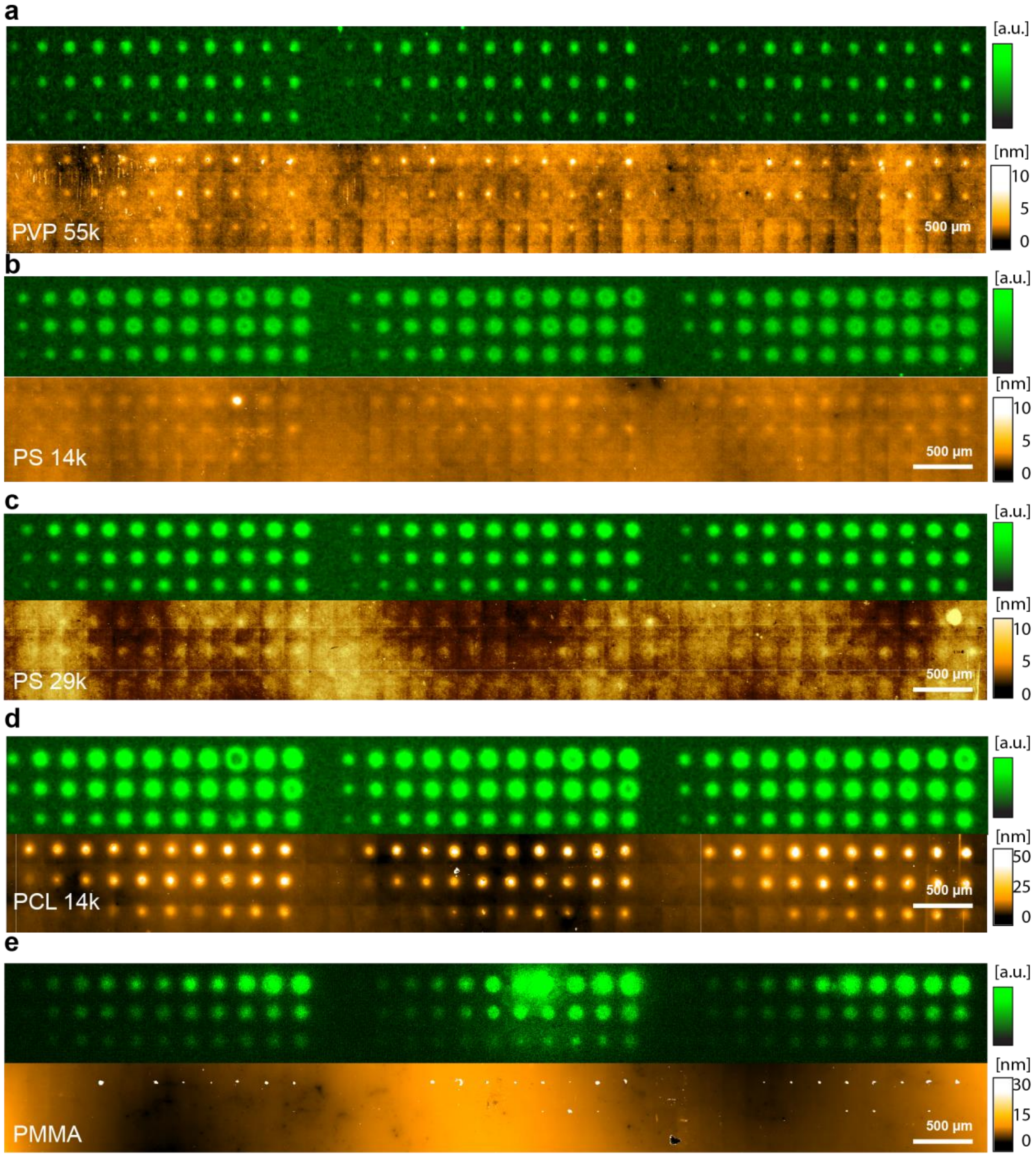

Figure S5. Various polymer transfers. Measured material transfer was inconsistent or too low for detection via VSI.

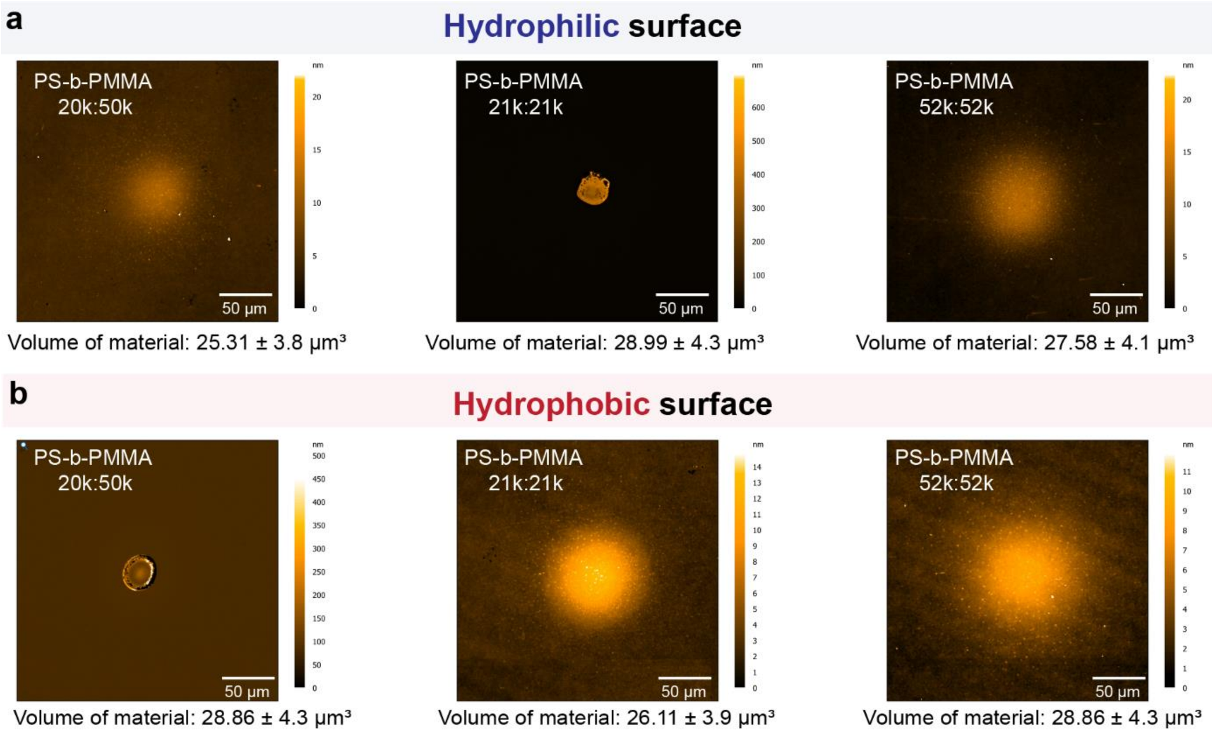

Figure S6. Individual VSI images of spots of the three block-co-polymers on (a) hydrophilic- and (b) hydrophobic surfaces, with the corresponding volume of material extracted from the topological measurement. All spots were prepared at 120 mW and 55 ms lasing parameters.

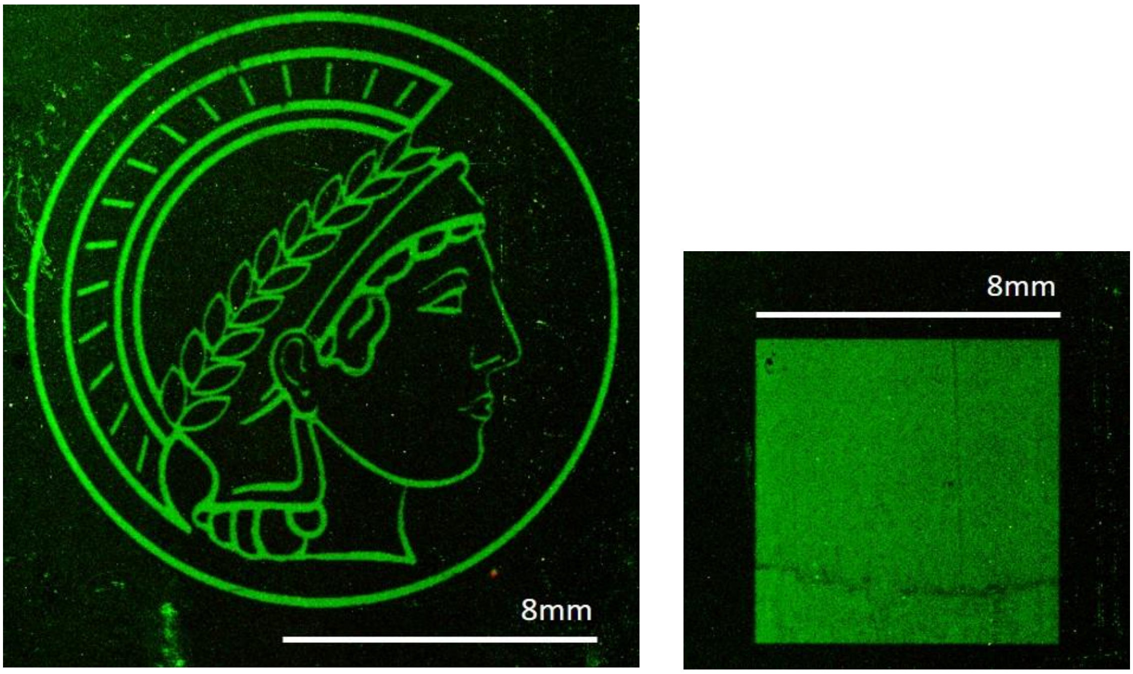

Figure S7. Fluorescence scan (auto-fluorescence) of transferred macroscopic polymer patterns. Minerva logo (left) and a square area (right), generated by transferring polystyrene onto a glass substrate.

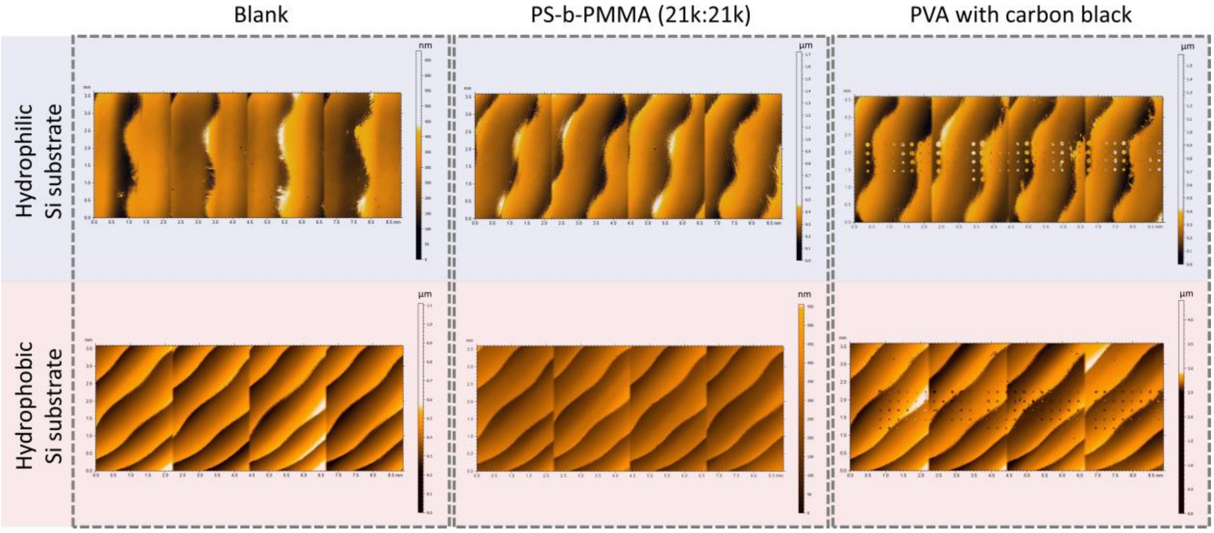

Figure S8. Vertical scanning interferometry (VSI) measurements of a hydrophilic (~ 5 nm thin oxide layer) and hydrophobic (~ 300 nm thick oxide layer) silicon substrate: blank, PS-b-PMMA (21k:21k), and PVA with carbon black spot pattern. Due to the strong light reflection and interference effects of these silicon surfaces, it is impossible to observe the polymer patterns on the substrate. For a qualitative result, carbon black was added to PVA as a contrast agent. This polar PVA formed larger spots on the hydrophilic Si substrate than on the hydrophobic Si substrate.

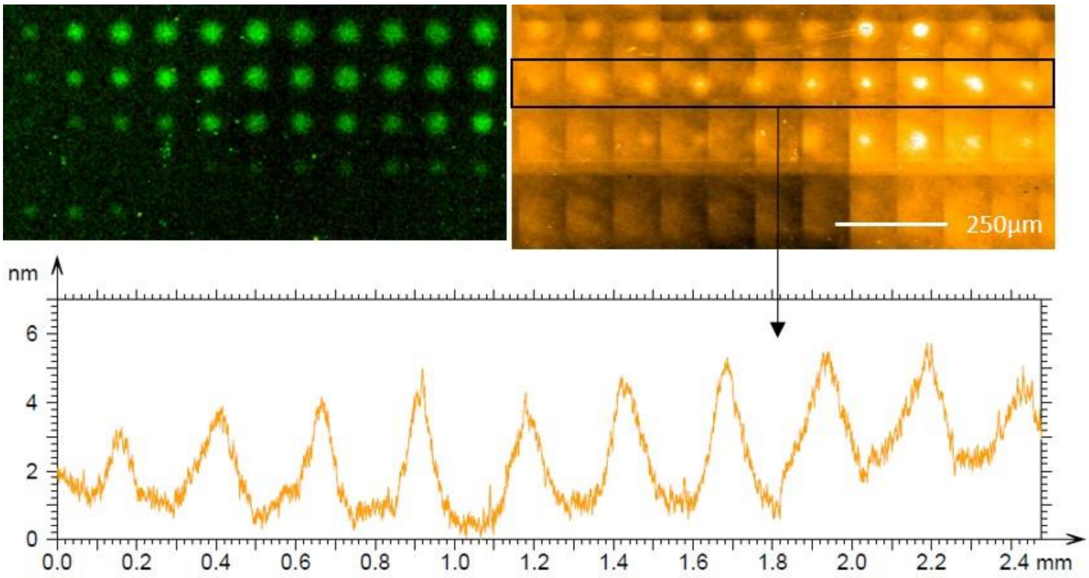

Figure S9. Fluorescence scan (left) and vertical scanning interferometry (VSI) measurement of spot gradient on a glass substrate, transferred from a 300 nm thin polystyrene donor layer.

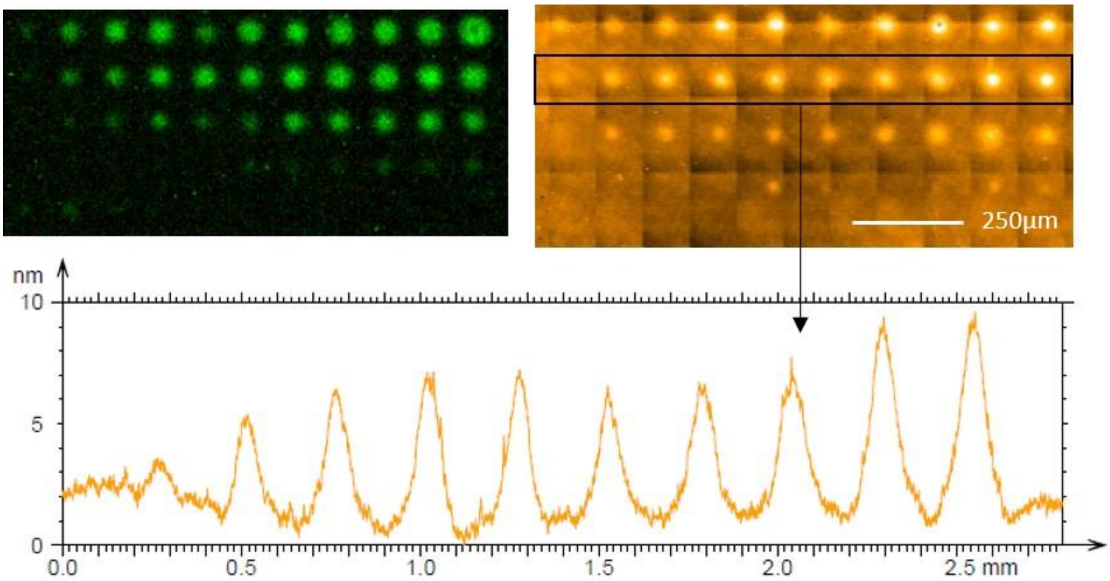

Figure S10. Fluorescence scan (left) and vertical scanning interferometry (VSI) measurement of a spot gradient on a glass substrate, transferred from a 700 nm thick polystyrene donor layer.

Table S1. Overview of additional thermoplastic polymers, transferred with the laser.

Formula	Name	Polymer	Weight	Vendor	T_g in [°C]	T_m (T_d(5%)) in [°C]
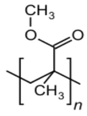	PMMA	Poly(methyl methacrylate)	M_n = 50,600	Sigma-Aldrich, Germany	118.2	(318)
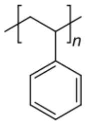	PS 14k	Polystyrene	M_n = 13,700	Sigma-Aldrich, Germany	97.6	(376)
	PS 29k		M_n = 29,300	Sigma-Aldrich, Germany	103.6	(378)
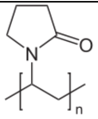	PVP 55k	Polyvinylpyrrolidone	M_w = 55,000	Sigma-Aldrich, Germany	181.2	(320)
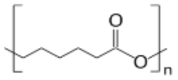	PCL 14k	Polycaprolactone	M_n = 10,000	Sigma-Aldrich, Germany	–60 [1]	53.8 (250)

References

1. Koleske, J. V.; Lundberg, R. D. Lactone polymers. I. Glass transition temperature of poly-ε-caprolactone by means on compatible polymer mixtures. *J. Polym. Sci. B Polym. Phys., 1969, 7, 795-807.*
